# Supplementary material for: Novel Nongenetic Murine Model of Hyperglycemia and Hyperlipidemia-Associated Aggravated Atherosclerosis
Source: Front Cardiovasc Med. 2022 Mar 8;9:813215. doi: 10.3389/fcvm.2022.813215 (PMC8957812; doi:10.3389/fcvm.2022.813215)
Supplement: Supplementary file 1 [file Data_Sheet_1.pdf]

Supplementary Material

__Content:__

Supplementary Table 1. Characteristics of mice cohort.

Supplementary Figure I. PD fed HGHCi mice showed higher mortality than HFD fed mice.

Supplementary Figure II. Determination of the external and internal elastic lamina, lumen area and media area.

Supplementary Figure III. Comparison of aortic plaque score and total vessel lumen area.

Supplementary Figure IV. Intraplaque hemorrhage is not increased in HGHCi HFD mice.

Supplementary Figure V. Hyperglycemia alone does not affect plasma cholesterol levels in mice fed a Paigen diet.

Major resources table

Supplementary Table 1. Characteristics of mice cohort.

High-fat diet	Body weight [g]	Liver weight [g]	Liver/body weight ratio
**12 weeks**			
Normal chow	31.8 ± 3.7[a]	1.5 ± 0.4[a]	0.04
Saline HFD	40.4 ± 2.3	2.8 ± 0.5	0.06
HCi HFD	39.1 ± 3.3	2.6 ± 0.5	0.07
HGHCi HFD	24.7 ± 0.9[b]	2.8 ± 0.2	0.11
LDLR KO HFD	36.3 ± 5.5	2.1 ± 0.6	0.05
**20 weeks**			
Normal chow	32.5 ± 2.8[a]	1.7 ± 0.1[a]	0.05
Saline HFD	42.6 ± 4.8	3.0 ± 1.1[b]	0.07
HCi HFD	45.3 ± 3.9[c]	4.1 ± 0.7[ac]	0.09
HGHCi HFD	27.3 ± 3.5[b]	2.7 ± 0.2[b]	0.10
LDLR KO HFD	39.2 ± 3.4	3.2 ± 0.7[c]	0.08
**Paigen diet**	**Body weight [g]**	**Liver weight [g]**	**Liver/body weight ratio**
**8 weeks**			
Saline PD	31.1 ± 1.2	2.0 ± 0.1	0.06
HCi Paigen	32.0 ± 3.5	1.7 ± 0.2	0.05
HGHCi Paigen	19.7 ± 3.1[b]	2.7 ± 0.1[b]	0.14
LDLR KO Paigen	27.9 ± 1.6[b]	1.6 ± 0.2	0.06

Supplementary Table 1. Data are shown as mean ± SEM and statistical comparisons between the groups were calculated using the one-way ANOVA and Sidak's posthoc test. [a] $p<0.05$ vs Saline HFD/PD, [b] $p<0.05$ vs HCi HFD/PD, [c] $p<0.05$ vs 12 weeks. HFD: high-fat diet; HCi: hyperlipidemia model; HGHCi: hyperglycemia + hyperlipidemia model; PD: Paigen diet

Supplementary Figure I. PD fed HGHCi mice showed higher mortality than HFD fed mice.

A

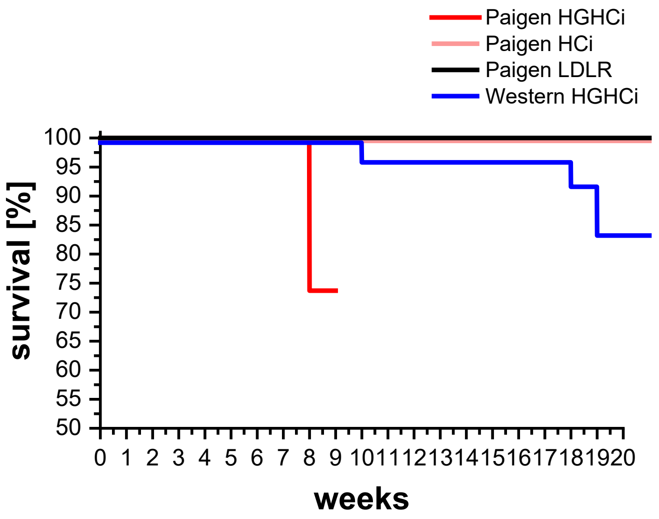

Supplementary Figure I. A: Kaplan-Meier survival curves of HGHCi, HCi and LDL receptor KO mice on Paigen diet compared to HGHCi mice on Western diet (high-fat diet, HFD).

Supplementary Figure II. Determination of the external and internal elastic lamina, lumen area and media area.

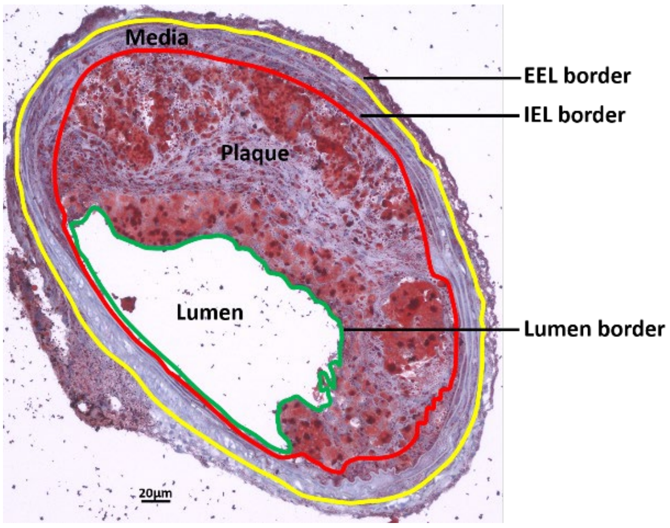

Supplementary Figure II. Representative image showing Oil-Red O stained BCA (**G**) for the measurement of the external elastic lamina (EEL, yellow line), internal elastic lamina (IEL, red line) and the lumen area (circled by a green line).

Supplementary Figure III. Comparison of aortic plaque score and total vessel lumen area.

A

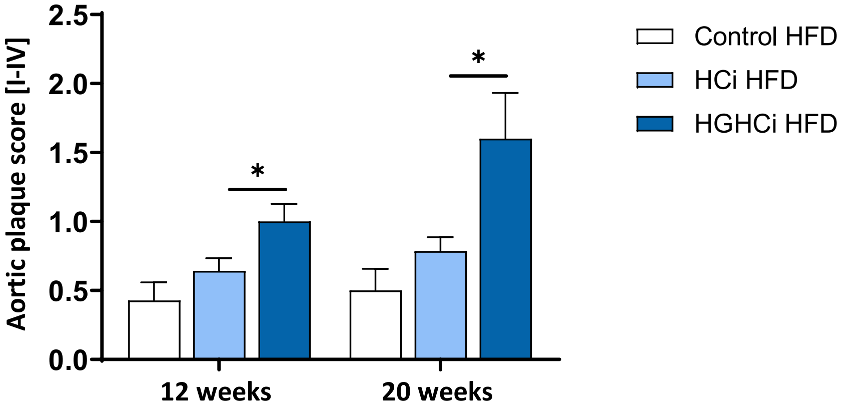

B

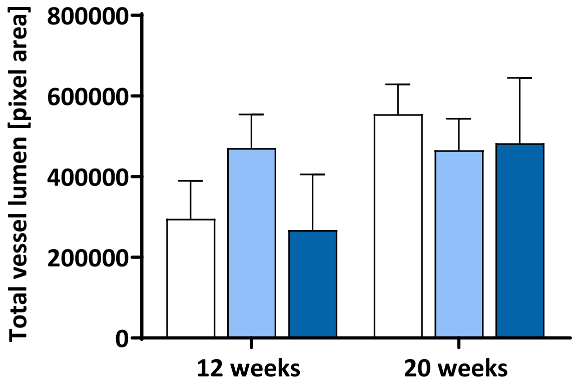

Supplementary Figure III. A: Aortic plaque score and total vessel lumen area [Pixel] of the truncus brachiocephalicus of HGHC and HCi mice on Western diet (high-fat diet, HFD) after 12 and 20 weeks. Aortic plaque score was determined as described below. 0= no lesions; 1= Lesions only in bifurcation; 2= like 1 + at least one long-stretch lesion; 3= like 1 + at least two long-stretch lesions; 4= like 1 + three to four long-stretch lesions

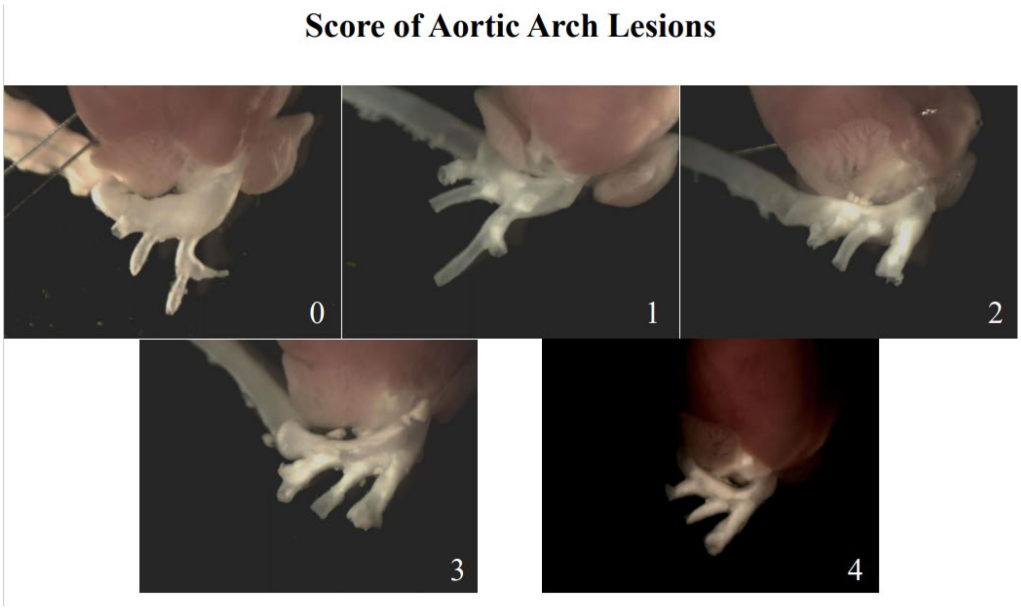

Supplementary Figure IV. Intraplaque hemorrhage is not increased in HGHCi HFD mice.

A

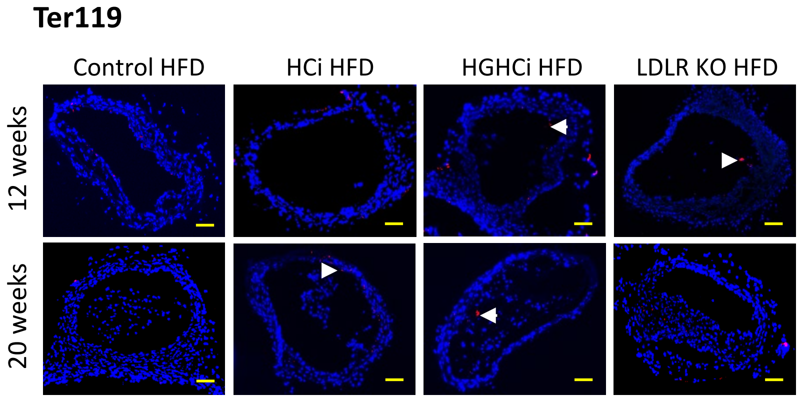

Supplementary Figure IV. A: Representative images showing immunofluorescence staining of truncus brachiocephalic arteries sections for hemorrhage marker Terr-119 (**A,** Terr-119= red; DAPI nuclear counterstain= blue, white arrows). Scale bar 200 µm. Control 12 and 20 weeks (N= 7), HCi 12 weeks (N= 5) and 20 weeks (N= 6), HGHCi 12 weeks (N= 7) and 20 weeks (N= 6).

Control HFD: Wild type mice without rAAV8-PCSK9^{D377Y} injection on high fat diet (HFD); HCi HFD: rAAV8-PCSK9^{D377Y} injection plus HFD (hyperlipidemic); HGHCi HFD: rAAV8-PCSK9^{D377Y} and streptozotocin injection and HFD (hyperlipidemic and hyperglycemic).

Supplementary Figure V. Hyperglycemia alone does not affect plasma cholesterol levels in mice fed a Paigen diet.

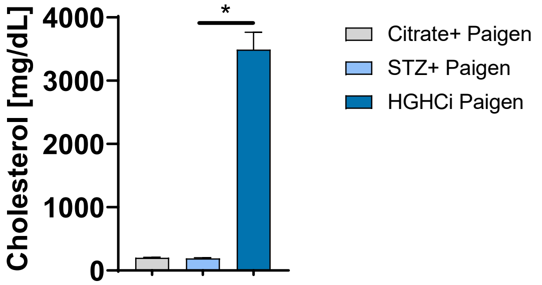

Supplementary Figure V. Hyperglycemia alone does not affect plasma cholesterol levels in mice fed a Paigen diet. Comparison of cholersterol levels in HGHCi mice (N=13), streptozotocin (STZ) treated mice (N=5) and citrate controls (N=3) fed with Paigen diet. Plasma cholesterol leves [mg/dL] were analyzed 12 weeks after induction of hypergylcemia with STZ.

Major Resources Table

In order to allow validation and replication of experiments, all essential research materials listed in the Methods should be included in the Major Resources Table below. Authors are encouraged to use public repositories for protocols, data, code, and other materials and provide persistent identifiers and/or links to repositories when available. Authors may add or delete rows as needed.

Animals (in vivo studies)

Species	Vendor or Source	Background Strain	Sex	Persistent ID / URL
Mouse	Charles River Laboratories	C57BL/6N	male	C57BL/6 Mouse | Charles River Laboratories. (criver.com)

Genetically Modified Animals

	Species	Vendor or Source	Background Strain	Other Information	Persistent ID / URL
**Parent - Male**	Mouse	The Jackson Laboratory	000664 C57BL/6J	B6.129S7-Ldlrtm1Her/J Stock No: 002207	https://www.jax.org/strain/002207
**Parent - Female**	Mouse	The Jackson Laboratory	000664 C57BL/6J	B6.129S7-Ldlrtm1Her/J Stock No: 002207	https://www.jax.org/strain/002207

Antibodies

Target antigen	Vendor or Source	Catalog #	Working concentration	Lot # (preferred but not required)	Persistent ID / URL
mLDLR	R&D System, United States	AF2255	1:200		https://www.rndsystems.com/products/mouse-ldlr-antibody_af2255
mβ-actin	Abcepta Inc., United States	AM1829B	1:10,000		https://www.abcepta.com/products/AM1829b-Beta-Actin-Antibody
Goat IgG HRP	Agilent Technologies, United States	P0449	1:1,000		https://www.agilent.com/store/en_US/LCat-SubCat3ECS_244797/Rabbit-Anti-Goat-Immunoglobulins-HRP
Mouse IgG HRP	Agilent Technologies, United States	P0447	1:10,000		https://www.agilent.com/en/product/immunohistochemistry/antibodies-controls/secondary-antibodies/goat-anti-mouse-immunoglobulins-hrp-(affinity-isolated)-153239
mMOMA-2	abcam, Germany	ab33451	1:100		https://www.abcam.com/monocyte--macrophage-antibody-moma-2-ab33451.html

DOI [to be added]

mCD68	abcam, Germany	ab125212	1:1,000		https://www.abcam.com/cd68-antibody-ab125212.html
Mouse alpha smooth muscle actin (mα-SMA)	abcam, Germany	ab124964	1:250		https://www.abcam.com/alpha-smooth-muscle-actin-antibody-epr5368-ab124964.html
Rat IgG Alexa Fluor 568	ThermoFisher, United States	A-11077	1:100		https://www.thermofisher.com/antibody/product/Goat-anti-Rat-IgG-H-L-Cross-Adsorbed-Secondary-Antibody-Polyclonal/
anti-rabbit HRP	Vector Laboratories, Inc., Unites States	MP-7801			https://vectorlabs.com/immpress-horse-anti-rabbit-igg-plus-polymer-kit-peroxidase.html

DNA/cDNA Clones

Clone Name	Sequence	Source / Repository	Persistent ID / URL

Cultured Cells

Name	Vendor or Source	Sex (F, M, or unknown)	Persistent ID / URL

Data & Code Availability

Description	Source / Repository	Persistent ID / URL

Other

Description	Source / Repository	Persistent ID / URL
BCA reagent	Perbio Science, Germany	
Vectashield mounting medium with DAPI	Vector Laboratories, United States	
Nitrocellulose membrane	Bio-Rad, USA	
Immobilon^{TM} western chemiluminescent HRP substrate	Merck, Millipore, United States	
Streptozotocin	Enzo Life Sciences, Germany	
Oil-Red O	Sigma-Aldrich, Germany	
Accu-chek test strips	Roche Diagnostics, Germany	
Accu-check glucometer	Roche Diagnostics, Germany	
Protease inhibitor cocktail	Roche Diagnostics, Germany	
Albumin fraction V	Carl ROTH, Germany	
Hematoxylin Gill II	Carl ROTH, Germany	

Acrylamide	Carl ROTH, Germany	
Agarose	Carl ROTH, Germany	
Aqueous mounting medium	ZYTOMED, Germany	
High fat diet	Ssniff, Germany	
PBS	Life Technologies, Germany	
Rompun	Bayer, Germany	
Ketamine	Beta-pharm, Germany	
